# Evaluation of Accuracy and Reliability of a Mobile Screening Audiometer in Normal Hearing Adults

**DOI:** 10.3389/fpsyg.2020.00744

**Published:** 2020-04-29

**Authors:** Angela Colsman, Gernot G. Supp, Joachim Neumann, Till R. Schneider

**Affiliations:** ^1^Department of Neurophysiology and Pathophysiology, University Medical Center Hamburg-Eppendorf, Hamburg, Germany; ^2^Sonormed GmbH, Hamburg, Germany; ^3^Independent Researcher, Hamburg, Germany; ^4^Mindo Software SLU, Barcelona, Spain

**Keywords:** audiometry, hearing test, air conduction, mobile application, automated audiometry, self-assessment, hearing threshold

## Abstract

Quantifying hearing thresholds via mobile self-assessment audiometric applications has been demonstrated repeatedly with heterogenous results regarding the accuracy. One important limitation of several of these applications has been the lack of appropriate calibration of their core technical components (sound generator and headphones). The current study aimed at evaluating accuracy and reliability of a calibrated application (app) for pure-tone screening audiometry by self-assessment on a tablet computer: Audimatch app installed on Apple iPad 4 in combination with Sennheiser HDA-280 headphones. In a repeated-measures design audiometric thresholds collected by the app were compared to those obtained by standardized automated audiometry and additionally test-retest reliability was evaluated. Sixty-eight participants aged 19–65 years with normal hearing were tested in a sound-attenuating booth. An equivalence test revealed highly similar hearing thresholds for the app compared with standardized automated audiometry. A test-retest reliability analysis within each method showed a high correlation coefficient for the app (Spearman rank correlation: rho = 0.829) and for the automated audiometer (rho = 0.792). The results imply that the self-assessment of audiometric thresholds via a calibrated mobile device represents a valid and reliable alternative for stationary assessment of hearing loss thresholds, supporting the potential usability within the area of occupational health care.

## Introduction

Hearing loss together with its negative personal and socio-economic consequences represents a serious health issue across cultures and around the globe. According to the World Health Organization (WHO, 2017) about 466 million people are already affected by a disabling hearing loss, while 1.1 billion young people (12–35 years) are at risk of hearing loss. The personal and socio-economic consequences of hearing loss are severe (Shield, 2006). In adults the consequences can range from mild impairments in the ability to communicate with others, pretending to hear, avoiding social situations (Thomas and Herbst, 1980; Kerr and Cowie, 1997) and increased stress (Hasson et al., 2011), to lower productivity, absenteeism, or loss of employment (Shield, 2006; Jung and Bhattacharyya, 2012). Hence, hearing loss cannot be confined solely as an individual problem, but it is rather directly linked to serious social and economic consequences (Mohr et al., 2000). The WHO (2017) underlines the importance of preventing and treating hearing loss as early as possible. For this purpose, mobile self-assessment audiometry represents an essential entry point to prevention and early treatment.

Hearing tests are necessary for the detection of hearing loss and are usually obtained by audiologists in clinical settings where the patient is seated in a sound-attenuating room (booth) during the testing. This procedure is time consuming, personnel-intensive and hence cost-intensive for the health care system. Therefore, audiometry is not implemented as a general assessment across the adult population, but rather performed selectively in individuals who are either suspected to have hearing loss or might be at risk of hearing loss (Davis et al., 1992; Gianopoulos et al., 2002; Shield, 2006). A cost-effective solution is beneficial to provide access for large parts of the population in order to detect early hearing loss. Self-assessment mobile audiometers might represent a possible solution for screening larger populations, provided that valid hearing thresholds are delivered (Margolis and Morgan, 2008). The intended use of the investigated app is to serve as a screening audiometer that could be applied in occupational health or in public institutions under supervision of trained personnel.

Automated audiometry in general has successfully shown to deliver hearing thresholds and several studies have provided evidence on the validity of hearing thresholds by automated audiometry comparable to manual audiometry (Mahomed et al., 2013). To date, the technical feasibility of self-assessment of hearing loss via audiometric applications, implemented on mobile devices, has been demonstrated by several studies (Bright and Pallawela, 2016), but only for a few self-assessment devices (uHear, EarTrumpet, hearTest, ShoeBox audiometry) published validation results are available (Szudek et al., 2012; Foulad et al., 2013; Handzel et al., 2013). Additionally, as an important limitation, only a minority of these studies reported on mobile audiometers with appropriately calibrated components (Thompson et al., 2015; Saliba et al., 2017; van Tonder et al., 2017).

The validity of a freely available iOS-based audiometric application (uHear) for hearing loss has been investigated in several studies (Szudek et al., 2012; Handzel et al., 2013; Khoza-Shangase and Kassner, 2013; Peer and Fagan, 2015; Abu-Ghanem et al., 2016). This device is connected to consumer earbud headphones, which are not calibrated with appropriate reference threshold values. The average difference in hearing thresholds compared to standardized automated audiometry was reported to be quite high: >8 dB HL (decibel hearing level) in a sound booth and >14 dB HL in a waiting room (Szudek et al., 2012). In a sample of elderly participants (>65 years) (Abu-Ghanem et al., 2016) found that the application overestimated hearing thresholds by up to 17 HL. Yet the authors note that it could serve as a useful screening device, especially for people with limited accessibility to audiometric facilities.

A self-assessment, tablet-based hearing test (ShoeBox audiometry) was validated in adults using a calibrated audiometric headphone revealing hearing thresholds within an acceptable range compared to conventional clinical audiometry (Thompson et al., 2015). The authors reported a deviation of hearing thresholds within 10 dB from those obtained by conventional audiometry and concluded that the device serves as a valid screening instrument.

For hearing tests, it is crucial that measurements of hearing levels gathered by different devices are comparable. Audiometric devices differ in their hardware components, i.e., in the tone-generating module and in the type of headphones. Headphones differ in frequency characteristics depending on their specific details of construction. Thus, the use of different types of headphones (circumaural, supra-aural, earbud, or insert headphones) can lead to large variations in amplitude of the test signal depending on their frequency response (Shojaeemend and Ayatollahi, 2018). Consequently, inaccuracies introduced by using non-standardized headphones, frequently applied in consumer products, can be resolved by applying calibrated, professional headphones designed for audiometry. Furthermore, adequate calibration requires the devices (sound generator and headphones) to be tested as a couple and calibration cannot be transferred to another device even when it is the same model.

Accurate interpretation of hearing thresholds requires calibration of the audiometric device, as comparability with established hearing tests cannot be ensured otherwise. To comply with this prerequisite a newly developed self-assessment tablet-based hearing test, calibrated with audiometric headphones, was validated and its reliability was tested in the current study. This app was developed for adults as a screening test. To evaluate the accuracy and reliability of the audiometric application we measured hearing-thresholds with the new app and compared these to standardized automated audiometry. For the comparison of thresholds, automated audiometry based on the Hughson-Westlake algorithm was applied, as this ensures a standardized procedure and high test-retest reliability. To answer the question whether differences in hearing thresholds between devices were sufficiently similar, an equivalence test (TOST-P) was computed for all frequencies (Rogers et al., 1993). Furthermore, we assessed the degree of reliability of the new app (and of the automated audiometer for comparisons) using within subject test-retest reliability analyses.

## Materials and Methods

### Study Design

The newly developed audiometric application was compared to standardized automated audiometry using a repeated-measures within-subject design. A conventional audiometer with automatic recording of hearing thresholds standardized according to DIN EN ISO 8253-1 (2011) was chosen as the reference (further denoted as AM). Thresholds were collected at eleven test frequencies ranging from 125 to 8000 Hz (see Table 2 for details). Test-retest reliability was computed on repeated hearing tests within subjects in both devices. The sample size was comparable to similar studies testing calibrated mobile audiometric devices (Yeung et al., 2013, 2015; Thompson et al., 2015).

### Participants

Sixty-eight participants were recruited either via local advertisements or from the database of the Department of Neurophysiology and Pathophysiology of the University Medical Center Hamburg-Eppendorf. Participants received remuneration for their participation (10 €/hour). Inclusion criteria for the participants were: normal hearing (according to self-report), no hearing aids, and an intact outer auditory canal. Participants were recruited so that the sample evenly covered the age range between 19 and 65 years (*M* = 42.1 years; *SD* = 13.74) and was roughly balanced according to gender (32 females, 36 males). Two participants had to be measured a second time on a separate day as their data was not stored correctly during the initial measurement.

### Equipment and Materials

Hearing thresholds were recorded automatically and independently for the two ears using pure-tone air-conduction applying the method of Hughson-Westlake following DIN EN ISO 8253-1 (2011). Hearing thresholds were recorded using (1) a conventional audiometer (AM: MAICO MA25 eIIID, Maico Diagnostics, Eden Prairie, MN, United States) and (2) a self-assessment, tablet-based audiometric application (app: Sonormed, Hamburg, Germany), which was executed on an iPad 4 (Apple Inc., Cupertino, CA, United States), equipped by default with a 32-bit digital-to-analog converter. The use of a 32-bit digital-to-analog converter is necessary to produce an adequate range of sound pressure levels (see Table 1 for ranges). The two devices are classified with medical class 1 and audiometer class 4. They were both equipped with Sennheiser HDA-280 headphones (Sennheiser, Wedemark, Germany), for which audiometric norms exist (Poulsen and Oakley, 2009). The supra-aural version of the HDA-280 was used in combination with the AM (Poulsen and Oakley, 2009) and the circumaural version of the same headphone (version HDA-280 CL, Parsa et al., 2017) was used with the iPad. The app and the specific combination of tone generator (iPad) and headphones were calibrated according to the IEC 60645 norm (IEC (60645)-1, 2017) by Audio-Ton GmbH (Hamburg, Germany) (Supplementary Material). The AM was calibrated according to the same norm (IEC 60645). The app was developed by Sonormed GmbH (Hamburg, Germany) and its core parts are marketed under the name “Audimatch” as an audiometric device.

**TABLE 1:** Specification of the minimum and maximum hearing level, which can be produced by the tone generator of the iPad using the app.

**Frequency (Hz)**	**Lower limit (dB HL)**	**Upper limit (dB HL)**
125	−20	75
250	−20	100
500	−20	110
750	−20	110
1000	−20	110
1500	−20	110
2000	−20	110
3000	−20	110
4000	−20	110
6000	−20	100
8000	−20	90

Both audiometric tests (app, AM) were conducted in a sound-attenuating room (Acoustair, Moerkapelle, Netherlands), which fulfilled the requirements of DIN EN ISO 8253-1 (2011) for air conduction audiometry. The manual for the setup of the app specifies the following prerequisites: a calm and distraction-free environment, comfortable room temperature, sufficient ventilation, and a rested state of the participant. The app automatically evaluates environmental noise and repeats measurement steps if the noise exceeds a defined level according to ANSI S3.1-1999 (ANSI S3 1-1999 (R2013), 2013). The investigator was outside the room during all tests but able to communicate with the participant via camera and intercom system. To compare test durations of the two devices, the duration of the hearing test with the AM was measured by the investigator using a stopwatch. The duration of the hearing test with the app was measured by the app itself. For both the app and the audiometer, the measurement of duration was started with the beginning of the familiarization phase (after the instruction) and ended with the response to the last tone.

### Procedures

The test session started with an explanation of the entire test procedure and written consent by the participants. An inspection of the outer ear and two screening tests for hearing according to Weber and Rinne were performed, before the main testing started.

The audiometric procedure of AM and app was technically similar (differences are listed in Table 2). The Hughson-Westlake procedure, which is an established method for automated audiometric tests (Carhart and Jerger, 1959; Rhebergen et al., 2015), was implemented in both devices: The hearing threshold at each frequency was successfully defined and accepted at the lowest level at which the participant detected two out of three ascending trials (DIN EN ISO 8253-1, 2011). The sound level was decreased by 10 dB if a positive response was given and increased by 5 dB if the participant did not indicate to hear the test tone.

**TABLE 2:** Comparison of audiometric procedures for the app (Audimatch) and the audiometer (MA25 eIII D).

**Feature**	**MA25 eIII D (AM)**	**Audimatch (app)**
Type of tone	Pure tone	Pure tone
Duration of tone	2s	1s
Response method	Signal detection, only “yes” responses	Alternative forced choice, “yes” or “no”
Possibility of response straight after beginning of tone	yes	no
Duration of response window	2s	unlimited, but pop-up window stating < < Choose “no” if you are not sure > > after 3s
Pause after response	2 – 3,6 s (random interval)	0.7s
Familiarization (right ear)	1000 Hz, presentation until double confirmation of threshold	1000 Hz, presentation until double confirmation of threshold
Familiarization level	Start value: 40 dB Level decrease for correct response: 10 dB Level increase for wrong response: 5 dB	Start value: 40 dB Level decrease for correct response: 20 dB Level increase for wrong response: 10 dB
Criterion for hearing-threshold	2 confirmations out of 3 presentations of the same ascending threshold at each frequency (shortened ascending method according to DIN EN ISO 8253-1)	2 confirmations out of 3 presentations of the same ascending threshold at each frequency (shortened ascending method according to DIN EN ISO 8253-1)
Ascending and descending step size (according to Hughson-Westlake algorithm)	5 dB HL up, 10 dB HL down	5 dB HL up, 10 dB HL down
Default intensity when changing frequency	10 dB decrease	20 dB increase
Default intensity when changing ear	30 dB	Hearing threshold from 1000 Hz in the right ear
Frequency [Hz] order right and left ear	1000, 1500, 2000, 3000, 4000, 6000, 8000, 1000, 750, 500, 250, 125, (1000)	1000, 1500, 2000, 3000, 4000, 6000, 8000, 1000, 750, 500, 250, 125
Re-Test at 1000 Hz	Yes	no
Procedure if deviation in re-test >10 dB HL	If difference at 1000 Hz ≤ 5 dB HL, change ear. Otherwise retest all frequencies until difference ≤5 dB HL.	not applicable
Class of medical device	1	1
Audiometer class	4	4

To reduce effects of learning, fatigue or attention the order of tests was counterbalanced either starting with the app or the AM. Both tests were conducted twice in alternate order (AM, app, AM, app).

#### Standard Automated Audiometer - AM

The investigator provided a standardized instruction according to DIN EN ISO 8253-1 (2011). Then the headphones were placed by the investigator. Before the main test started a familiarization procedure at 1 kHz was conducted on the right ear. Following the familiarization, the order of the eleven test frequencies was: 1 to 8 kHz, again 1 kHz and finally down to 125 Hz. Pure tones with a fixed duration of 2 s were used as test tones. Responses were registered via a hand-held response button. Participants were instructed to press the button only when they heard a tone. The tone was interrupted as soon as a response was given. If no response was given within 2 s after tone offset, the next tone was presented. The inter-stimulus interval varied from 2 to 3.6 s. When a threshold was determined for one frequency, the sound pressure level was increased by 10 dB for the next frequency. Before changing to the opposite ear, the hearing threshold at 1 kHz was tested again. If the deviation at 1 kHz was 5 dB or less, the more sensitive threshold was taken. If the threshold deviated more than 5 dB from the previously measured value, the test sequence started again until deviations were not larger than 5 dB. The default intensity of the start frequency at the opposite ear was 30 dB HL.

#### Mobile Screening Application - App

A standardized instruction was implemented in the app as a startup-wizard, which each participant had to execute. To guarantee voluntary participation, the user could terminate the test at any time. The startup-wizard included the following: a reminder to take a comfortable seating position and to avoid noise, e.g., through unnecessary movements; a reminder to draw full attention to the test. The headphones were placed by the users themselves. The correct left/right positioning of the headphones was tested by presenting test tones to each ear. Instructions on the screen requested the user to take off glasses, head covers, and jewelry that could interfere with the correct placement of the headphones.

A familiarization procedure at 1 kHz was conducted before the main test. At all times pure tones with a fixed duration of 1 s were used as test tones and it was not possible to respond before the end of the tone. Following the tone, participants had to respond to the question “Did you hear a tone?” with “yes” or “no.” The choice screen initiated the response interval. If the response time lasted longer than 3 s, the following message was presented: “choose *no* if you are not sure.” The inter-stimulus interval was fixed to 0.7 s. When a threshold was determined for one frequency, the sound pressure level was increased by 20 dB for the subsequent frequency. Following the familiarization, the order of the eleven test frequencies was: 1 to 8 kHz, again 1 kHz and finally down to 125 Hz. For the opposite ear the threshold of the first ear at 1 kHz was taken as the starting level.

At the end of the procedure, app test results were presented to the participants in five grades according to the criteria of the WHO (2018): normal hearing (≤25 dB HL), mild (26–40 dB HL), moderate (41–60 dB HL), severe hearing loss (>60 dB HL), for each ear and for four frequencies separately: 500, 1000, 2000, and 4000 Hz. In case of hearing loss of more than 25 dB HL at least in one ear and in one frequency, or in case of any doubts on subjective hearing, the app recommends to consult a hearing health professional.

#### Total Duration of Audiometric Testings

The total duration of the four audiometric tests was approximately 60–80 min, including the instruction and breaks, which were scheduled after each audiometric test. During the breaks with self-determined duration, participants had the possibility for refreshment. The duration of the breaks did not exceed 5 min. All test sessions were conducted within the timespan from 9 a.m. to 6 p.m. Resulting hearing thresholds were explained to participants after the complete test-procedure with reference to hearing thresholds appropriate for age and gender according to ISO 7029 (Eyken et al., 2007).

### Data Analysis

The main goals of the analysis were (1) to evaluate the accuracy of the app, i.e., the agreement of hearing thresholds recorded by the app compared to the AM, and (2) to assess the reliability of the app, i.e., evaluating the degree of precision of measurements of the app in a test-retest analysis. The first goal was addressed by testing whether differences in hearing thresholds between devices fall within an acceptable range of deviation to be expected for audiometric measurements using an equivalence test and furthermore by calculating the correlation between the audiograms of app and AM thresholds. The second goal was evaluated by test-retest reliability as estimated by correlating hearing thresholds of the same participants and the same device (correlations within app and within AM thresholds).

Data analysis was performed using R and R Studio (R Version 3.3.3, R Studio Version 1.0.153). For the comparison of thresholds, ears of participants were treated as independent units of observation, as hearing loss can occur in one ear independently from the other one. A significance level of *p* < 0.05 was defined for all statistical tests.

#### Accuracy

We examined the similarity of hearing thresholds obtained with the app and the AM. The most frequently used method to evaluate the accuracy of measurement instruments is the report of average differences and absolute average differences including standard deviations (Mahomed et al., 2013). In addition to these descriptive statistics, an inferential statistical approach was used to test the accuracy of the app. A two one-sided paired t-test procedure (TOST-P, Rogers et al., 1993) was computed as an equivalence test, in order to test whether hearing thresholds obtained with the app and the AM are sufficiently equivalent. The acceptable range of deviation was defined by standards described in ISO 8253. Equivalence tests were developed in social sciences for research questions asking rather for the equivalence of impact of two treatments than for their difference. With the TOST-P procedure two H0 hypotheses are proposed, assuming that the mean difference of hearing thresholds falls outside a certain *a priori* defined range (Mara and Cribbie, 2012):

H0_1_: μ_1_−μ_2_ ≥ δ and H0_2_: μ_1_−μ_2_ ≤ −δ

With μ_1_ and μ_2_ being the expected mean value in the population, and δ and −δ being the upper and lower boundary of the accepted range of deviation between two devices, denoted as the interval of equivalence (Mara and Cribbie, 2012). The test will only be significant, if both null hypotheses (H0_1_, H0_2_) are rejected. In this case the H1, i.e., the mean value lies within the accepted range of deviation, will be accepted. The critical *t*-value for the H0_1_ is calculated with the mean values of hearing thresholds in the AM (M1) and the app (M2) according to Mara and Cribbie (2012):

$$t\,2=\frac{M_{1}-M_{2}-\delta }{\frac{s\,D\,i\,f\,f}{\left(n-1\right)}}$$

and *t*-value for H0_2_ according to:

$$t\,2=\frac{M_{1}-M_{2}-\left(-\delta \right)}{\frac{s\,D\,i\,f\,f}{\left(n-1\right)}}$$

with s_Diff_ being the standard deviation of the differences and n the sample size. Hence H0_1_ would be rejected if t_1_ ≤ −t_α_, (_n–1_) and H0_2_ would be rejected if t_2_ ≥ t_1–α_, (_n–1_), respectively, with alpha being the significance level. As 11 tests (one for each frequency) were performed, testing the hypothesis that the devices did not differ in accuracy, a Bonferroni correction for multiple comparisons was applied by dividing the significance level by the number of tests: *p* = 0.05/11 = 0.0045. The Bonferroni correction reduces the probability of false positives. Of note, with the TOST-P this is the probability that the test erroneously assumes that the two devices do not differ in their thresholds, although they do differ.

The interval of equivalence was defined as the maximally tolerable deviation (δ and −δ) due to the measurement uncertainty according to DIN EN ISO 8253-1 (2011). The calculation of the measurement uncertainty yielded a maximum tolerable deviation of 10 dB HL in all frequencies from 125 to 4 kHz, and 13 dB HL above 4 kHz. Hence a deviation of ± 10 dB HL (and ± 13 dB HL for 6 and 8 kHz) was chosen as the boundary values for the interval of equivalence in the TOST-P. The measurement uncertainty considers that audiometric data varies due to several aspects of the test setting, the participant, the nature of repetition, and expected values close to the normal hearing threshold.

As a second approach to quantify the agreement of the measured values, correlation coefficients between hearing thresholds obtained by the two devices (app and AM) were computed. A Spearman rank correlation was calculated, because hearing thresholds were not normally distributed.

#### Test-Retest Reliability

The correlation of hearing thresholds obtained with the same device and same participants at different points in time, the test-retest reliability coefficient (*r*_tt_), can be considered as the method’s degree of precision. It is assumed that the true values of participants do not differ between measurements (Moosbrugger and Kelava, 2012). Again, Spearman rank correlations were calculated, because hearing thresholds were not normally distributed.

#### Test Duration

To examine a potential difference in test duration between the two devices, the time intervals to complete the respective audiometric measurements were compared and statistical significance of the difference was tested with a two-sided paired *t*-test. To assess whether the test duration within the app differed between the first and the second run, a two-sided Wilcoxon signed rank test was computed, as duration differences between t1 and t2 were not normally distributed.

## Results

According to standardized automated audiometry, 66 of 68 participants had normal hearing defined by the WHO criteria, i.e., pure-tone average (PTA) of 0.5, 1, 2, 4 kHz: Normal hearing (≤25 dB HL), mild (26–40 dB), moderate (41–60 dB), severe (61–80 dB), or profound hearing loss like deafness (>81 dB) on the better hearing ear. Two participants had mild hearing loss with ∼35 dB HL on the better hearing ear. This result was obtained identically by the app and the AM. This decision of using the WHO criteria for hearing loss bears the potential disadvantage that unilateral hearing loss, or hearing loss in single frequencies is missing. Further results are very detailed and contain all single frequencies and both ears. The following results are reported for 136 ears of 68 participants.

### Accuracy

**FIGURE 1:**
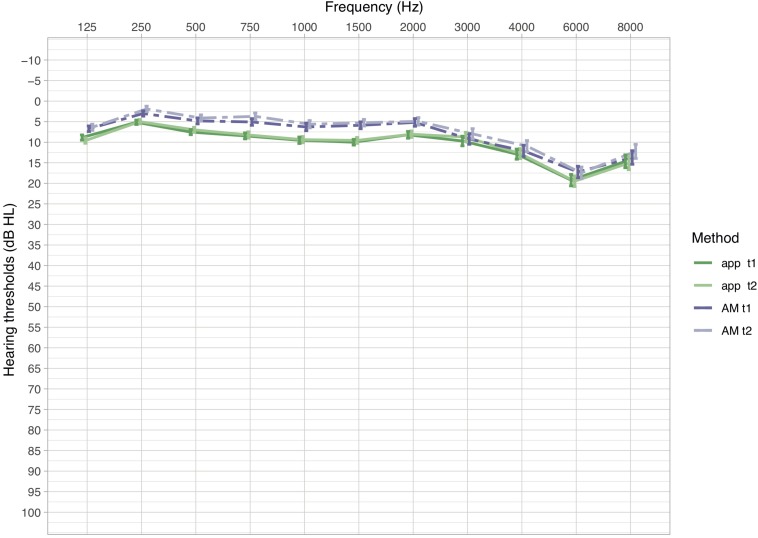
Mean hearing thresholds in dB HL (±SEM) of measurements in the app and the AM for the first and second measurement (t1 and t2). Dashed lines indicate hearing thresholds of the AM, solid lines of the app. Lower dB HL values in the audiogram imply lower hearing thresholds, hence better hearing.

Descriptive analyses of the mean hearing levels (in dB HL) revealed that thresholds measured with the app were slightly higher than thresholds measured with the AM (Figure 1). This relationship was observed in all frequencies. Mean differences in hearing levels between the app and the AM ranged between −4.12 and −0.48 dB (AM -app) with standard deviations between 3.49 and 6.7 dB across the frequency spectrum, while mean absolute differences ranged between 3.27 and 4.96 dB with standard deviations between 2.93 and 4.7 dB (Table 3).

**TABLE 3:** Results of comparisons between App and AM: mean differences and mean absolute differences (dB HL) of hearing thresholds, in each frequency across all ears.

**Frequency [Hz]**	**Mean diff. (AM-App) [dB HL]**	**SD**	**Mean abs. diff. [dB HL]**	**SD**
125	−2.132	6.135	4.706	4.462
250	−2.132	4.200	3.382	3.271
500	−2.721	3.489	3.309	2.933
750	−3.382	3.746	3.824	3.291
1000	−3.235	4.851	4.338	3.888
1500	−4.118	4.709	4.853	3.941
2000	−3.015	5.453	4.485	4.315
3000	−0.478	4.630	3.272	3.299
4000	−0.809	5.027	3.456	3.728
6000	−1.985	6.033	4.265	4.696
8000	−0.919	6.700	4.963	4.574

*SD = standard deviation.*

**FIGURE 2:**
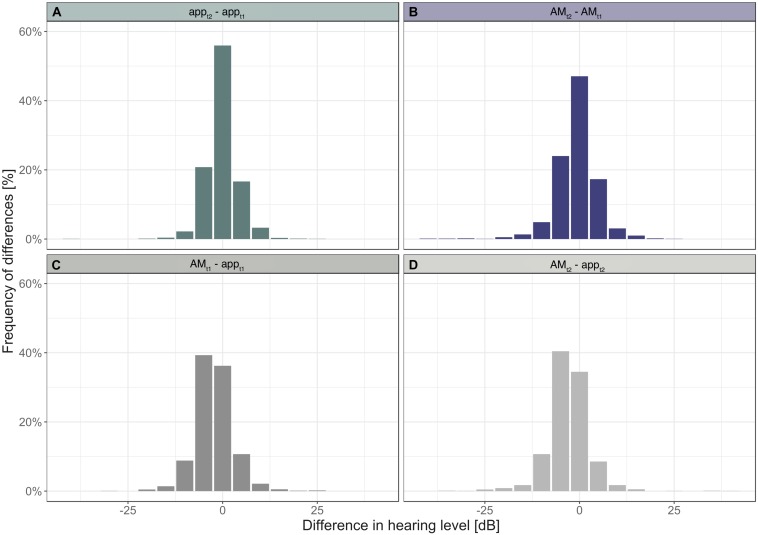
Histograms of differences in hearing levels within devices **(A,B)** and between devices **(C,D)**.

The distribution of differences between hearing thresholds of the app and the AM across subjects show that at t1 approximately 97% of all differences fall within the range of ± 10 dB HL and at t2: 96% (Figure 2C). These values indicate that most differences lie within the interval of equivalence.

**FIGURE 3:**
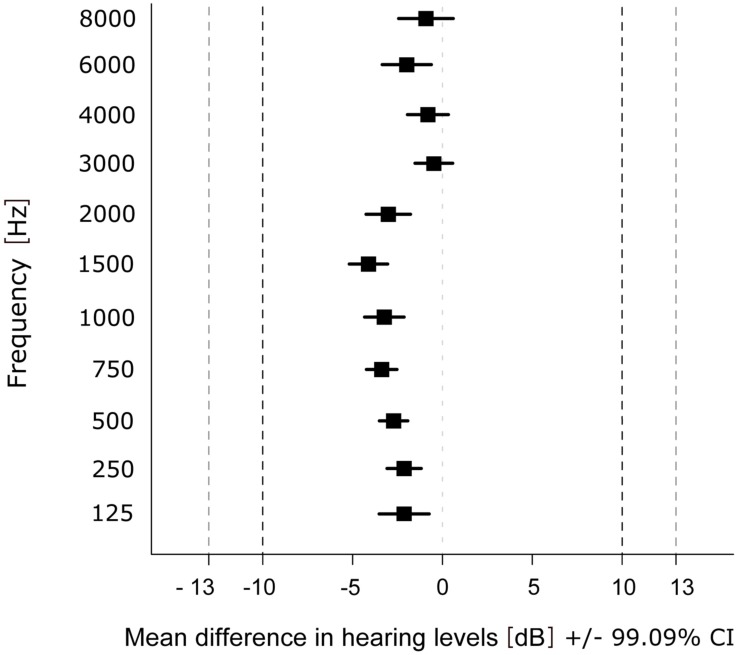
Mean difference in hearing levels within each frequency over participants and ears. Dashed lines show upper (+10 dB HL) and lower (–10 dB HL) boundary of the interval of equivalence (and ±13 dB HL for 6000 and 8000 Hz).

For the statistical evaluation of similarity of the hearing thresholds obtained with the app and the AM, the data was analyzed between devices using the first run (t1) only. The equivalence test revealed that differences in hearing thresholds fall into the interval of equivalence for all frequencies (Figure 3). The smallest difference between the two devices was observed at 3000 Hz (*M*_Diff_ = −0.48; *SD* = 4.63, *t*(135) = 23.984, *p* < 0.001) and the largest at 1500 Hz (*M*_Diff_ = −4.12; *SD* = 4.71, *t*(135) = 14.566, *p* < 0.001). Results of the TOST-P for all frequencies can be found in Table 4.

**TABLE 4:** Results of comparisons between devices: mean differences (dB HL) of hearing thresholds, *p*-values and 99.09% CI for the TOST-P in each frequency across all ears.

**Frequency [Hz]**	**Mean diff [dB HL]**	***t* (135)**	**p**	**99.09% CI**
125	−2.132	14.955	<0.001	[−3.525, −0.74]
250	−2.132	21.845	<0.001	[−3.086, 1.179]
500	−2.721	24.331	<0.001	[−3.512, −1.929]
750	−3.382	20.603	<0.001	[−4.233, −2.532]
1000	−3.235	16.263	<0.001	[−4.336, −2.134]
1500	−4.118	14.566	<0.001	[−5.187, −3.049]
2000	−3.015	14.939	<0.001	[−4.252, −1.777]
3000	−0.478	23.984	<0.001	[−1.529, 0.573]
4000	−0.809	21.324	<0.001	[−1.95, −0.332]
6000	−1.985	21.291	<0.001	[−3.355, −0.616]
8000	−0.919	21.028	<0.001	[−2.44, 0.602]

**FIGURE 4:**
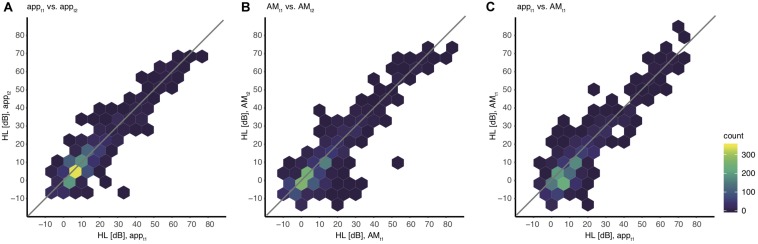
Association between hearing levels within and between devices including all values. The color indicates the absolute number of thresholds for all individual ears and all frequencies. Note that single outliers (one ear, one frequency) can appear already as a dark blue hexagon. **(A)** app_t1_ vs. app_t2_, **(B)** AM_t1_ vs. AM_t2_, **(C)** app_t1_ vs. AM_t1_.

Furthermore, the hearing thresholds of the app were correlated with those of the AM to test the agreement of the hearing thresholds collected by the app. The partial Spearman rank correlation between the hearing thresholds of all frequencies obtained at t1 was rho_APP, AM_ = 0.777 (*p* < 0.001). A scatter plot of the hearing thresholds indicates a strong association between the two devices (Figure 4C).

### Test-Retest Reliability

For an adequate interpretation of the results obtained by the analysis of the validity, the reliability of the app needs to be quantified explicitly. Therefore test-retest reliability was computed within both devices. A partial Spearman rank correlation, corrected for frequency, resulted in a test-retest correlation coefficient of rho_tt, app_ = 0.829 (*p* < 0.001) for measurements with the app and rho_tt, AM_ = 0.792 (*p* < 0.001) for measurements with the AM (Figures 4A,B).

The distribution of differences in thresholds between the first and second measurement obtained with the app revealed that 93% of threshold values differed by 5 dB or less (Figure 2A). With the AM 88% of threshold values differed by 5 dB or less (Figure 2B). A difference of 0 dB was observed for 56% of threshold values when tested with the app, and 47% when tested with the AM.

Hearing thresholds within devices show a strong correlation between the first and the second session, also when considering single frequencies (Table 5). Correlation coefficients were highest in the high frequencies and lowest at 125 Hz within both devices. Test-retest reliability values within frequencies (Table 5) in the app vary from rho_(tt)125_ = 0.728 (*p* < 0.001) at 125 Hz up to a very high correlation at 8000 Hz rho_(tt)8000_ = 0.919 (*p* < 0.001). Test-retest reliability values within frequencies in the AM vary from rho_(tt)125_ = 0.614 (*p* < 0.001) at 125 Hz up to a very high correlation at 8000 Hz rho_(tt)8000_ = 0.902 (*p* < 0.001).

**TABLE 5:** Results of test-retest reliability analyses within device and test frequency.

**Frequency [Hz]**	**Device**	**rho_t1,t2_**	**p**	**95% CI**
125	app	0.728	<0.001	[0.638; 0.799]
	AM	0.614	<0.001	[0.497; 0.709]
250	app	0.747	<0.001	[0.663; 0.814]
	AM	0.74	<0.001	[0.652; 0.807]
500	app	0.831	<0.001	[0.770; 0.877]
	AM	0.781	<0.001	[0.706; 0.839]
750	app	0.763	<0.001	[0.683; 0.826]
	AM	0.798	<0.001	[0.728; 0.852]
1000	app	0.75	<0.001	[0.665; 0.815]
	AM	0.719	<0.001	[0.627; 0.792]
1500	app	0.750	<0.001	[0.666; 0.815]
	AM	0.736	<0.001	[0.647; 0.804]
2000	app	0.84	<0.001	[0.782; 0.883]
	AM	0.754	<0.001	[0.670; 0.818]
3000	app	0.843	<0.001	[0.786; 0.886]
	AM	0.853	<0.001	[0.799; 0.893]
4000	app	0.899	<0.001	[0.861; 0.927]
	AM	0.868	<0.001	[0.82; 0.904]
6000	app	0.857	<0.001	[0.805; 0.896]
	AM	0.825	<0.001	[0.763; 0.872]
8000	app	0.919	<0.001	[0.888; 0.941]
	AM	0.902	<0.001	[0.865; 0.929]

### Test Duration

To assess whether durations to complete the test with the app and the AM are comparable, the duration of each audiometric test was measured. For both devices the time interval included the familiarization phase, but not the instruction. Due to transmission errors, durations of four subjects were lost in the data set of the app. The median of durations at t1 to complete the test with the app was *MD* = 12.38 min (IQR = 2.179) and with the AM it was MD = 16.51 min (IQR = 3.45). Minimum and maximum times to complete the test with the app were 10.05 min and 19.73 min and with the AM were 13 and 28.5 min. The time to complete the hearing test with the app was significantly shorter than with the AM at t1: The average difference was *M*_Diff_ = −4.27 min (95% CI = [−4.866; −3.676]), *t*_(63)_ = −14.335, *p* < 0.001.

To assess whether a learning effect had occurred in the use of the app, reflected in a shorter duration of the second testing, a two-sided Wilcoxon signed rank test was computed. The duration with the app at t2 was *MD* = 11.98 min (IQR = 2.566) and with the AM it was *MD* = 16.23 min (IQR = 3.49). Within audiometric tests no significant differences between t1 and t2 durations were observed (*MD* = −0.4 min; IQR = 1.758), *V* = 0.713, *p* = 0.096. For this comparison the duration values of seven participants were lost, due to the transmission error of the app data.

## Discussion

The results of the current validation study revealed similar audiometric thresholds for the mobile screening audiometer application compared to standardized automated audiometry. Differences between the audiograms of both devices ranged within a clinically acceptable range as indicated by an equivalence test. The convergence of both audiograms revealed by each device was demonstrated by the correlation of hearing thresholds. The degree of reliability of the mobile screening audiometer was quantified by a test-retest reliability analysis. The resulting correlation coefficient revealed a high degree of precision for the app and showed similar levels compared to standardized automated audiometry.

### Evaluating Accuracy and Reliability

The first aim of the present study was to investigate the validity of the mobile screening audiometer. To that end, we compared the hearing thresholds of the app with hearing thresholds of a standard automated audiometer as used in clinical context. An equivalence test (TOST-P) indicated a good agreement between the auditory thresholds of both devices. The test revealed that mean differences between app and AM are significantly smaller than the limits of uncertainty that are common to audiometric procedures. Therefore, we concluded that the hearing thresholds gathered with the app seem to be within a clinically acceptable range of deviation compared to hearing thresholds obtained with a standard automated audiometer. Agreement of thresholds is also supported by the significant correlation between hearing thresholds of the two devices. In the current study 97% of detected thresholds were within the clinically acceptable range of difference (10 dB HL) compared to standardized automated audiometry. Compared to other studies investigating the validity of the mobile audiometric applications, the corresponding percentage of values within the acceptable range in the current study is comparable (Thompson et al., 2015; van Tonder et al., 2017) or even higher (Szudek et al., 2012; Foulad et al., 2013; Khoza-Shangase and Kassner, 2013).

Mobile audiometric devices without calibrated headphones show larger differences in hearing thresholds compared with conventional audiometry (Szudek et al., 2012; Foulad et al., 2013; Khoza-Shangase and Kassner, 2013). Several studies investigating the validity of non-calibrated mobile audiometric devices reveal average differences in hearing thresholds >14 dB compared to conventional audiometry (Khoza-Shangase and Kassner, 2013) and the percentage of thresholds ranging within 10 dB of the conventional audiogram at 67% when measured in a sound booth (Szudek et al., 2012). Foulad et al. (2013) investigated the validity of a self-assessment mobile application (EarTrumpet) with different iOS devices. However, the system used earbud headphones calibrated with a non-standardized procedure. Further studies, which investigated the validity of mobile hearing tests, either tested applications which were not designed for self-assessment (hearScreen and AudCal) (Swanepoel et al., 2014; Larrosa et al., 2015) or did not use calibrated headphones (Kam et al., 2012; Corry et al., 2017). Thus, they are not directly comparable to the audiometric application tested in the current study.

Applications which were tested with calibrated headphones resulted in much higher accuracies than the uncalibrated devices (Yeung et al., 2013; Thompson et al., 2015; Saliba et al., 2017; van Tonder et al., 2017). In fact, the percentage of thresholds within 10 dB of the corresponding conventional audiogram ranged between 91 and 97%: the result of 97% was reported by Thompson et al. (2015), 94.4% by van Tonder et al. (2017), and 91.1% (EarTrumpet) and 95.5% (Shoebox audiometry) by Saliba et al. (2017), which all used calibrated devices. These accuracy values are in the same range as the accuracy values of the app under investigation here.

Most mobile audiometric devices suffer from a limited range of sound intensities due to the technical limitations of headphones or the acoustic converter. At the lower boundary of sound intensities other studies report a technical limitation at 10 dB HL (van Tonder et al., 2017) or 15 dB HL (Thompson et al., 2015) leading to floor effects in normal hearing individuals. The app under investigation can produce sound intensities as low as −20 dB HL and up to 110 dB HL (see Table 1), which reduces the probability of floor or ceiling effects.

The second aim of the study was to evaluate the degree of reliability of measurements of the app by performing a test-retest analysis. This analysis revealed a high degree of precision for the app with slightly higher correlation coefficients compared to the audiometer. Only the evaluation of one other computerized and self-assessment hearing test (Kam et al., 2012) was reported to have higher retest-reliability values for the mobile application (intra-class correlation = 0.95) than the ones found in the current study.

Taken together, the findings of the present study support the assumption that the app under investigation showed acceptable values of accuracy and high reliability which are in the range of automated clinical audiometers (Mahomed et al., 2013). The device could serve as a valid screening audiometer. These results extend findings of previous research on mobile self-assessment audiometry in terms of reliability and accuracy. Our results further support the necessity that for the self-assessment of hearing thresholds the use of appropriately calibrated devices according to ISO standards is beneficial and helps to obtain levels of accuracy and reliability. The potential areas of usage for mobile screening tests are versatile and include, but certainly are not constrained to, occupational health care and public health institutions. Mobile audiometric devices could provide a low-threshold access to hearing tests within companies that offer this health-related service to their employees. In addition, such a screening device might be integrated in awareness campaigns addressing hearing health and prevention within occupational health care. Although screening results cannot substitute a detailed audiometric evaluation of a medical specialist, the result can motivate a person to visit a medical specialist in order to receive a diagnostic clarification and professional assessment.

### Test Duration

The duration to complete an audiometric test with the app was on average 4.27 min shorter than with the AM, pointing to a potential time-saving advantage for the investigator and reducing task-related fatigue for the participant. Three main features, which could affect the duration of the test, were different between the app and the audiometer: the duration of the test tones, the interval between response and the next tone, and the type of question the participants had to answer (press a button only when a tone is present vs. press different buttons for tone present/not present). These three differences could have led to faster detection of thresholds without strongly affecting the validity of the new audiometric device, as differences between both devices were within the limits (97% of thresholds were within 10 dB difference).

A learning effect for the execution of the app was not detected, as test durations in the first compared to the second run were similar. Similar durations for both runs also indicate that the instructions for the app were sufficiently well understood before the first run. Also, an effect of fatigue seems unlikely, given similar test durations at the beginning and the end of the whole testing session.

### Limitations of the Study

Test sessions were conducted in a sound-insulated booth and it is therefore not evident whether results can be compared to the measurement of hearing thresholds in a noisy surrounding, like in a standard office. Thus, field studies with environmental noise could provide more insight on the accuracy and validity of the audiometric thresholds gathered by the app, for example in a waiting room of an otolaryngologist. Importantly, a validation with audiologically impaired patients would be necessary for the estimation of sensitivity and specificity of the app for clinically relevant hearing loss.

The audiometric application was designed for self-assessment. However, even though the whole audiometric procedure can be performed by the participant, the system is not intended for the use in private homes, outside the range of a trained person, as special headphones are needed and regular calibration of the iPad/headphone combination is a prerequisite. Apart from the calibration of the system, which has to be performed by a specialized company, this audiometric screening test can be operated by the user. Some supervision by trained personnel is helpful when starting the app, but it does not require the guidance of a health care professional. This stands also in contrast to the operation of the automated audiometer (which was used for comparison) for which the placement of the headphones and the instruction of the participants had to be done by a trained person.

A further limitation of the study is concerning the sampling method. Gender and age range are well-known factors which influence hearing thresholds (Vaz et al., 2013). To avoid biases due to an overproportionate representation of these specific attributes, we used a sampling method, which allowed to collect data from a more representative sample than simple random sampling. This method accepts the consequence that the recruiting is not completely random. The proportion of males and females in the sample was balanced, and age was uniformly sampled across the age range of the study.

Two authors of the current article were involved in the development of the app-based mobile hearing test which was evaluated in this study. This fact was disclosed before the start of the study so that a potential influence on the design of the study, data collection or the rational of data analysis could be contained beforehand.

## Conclusion

Given the experimental settings of the present study, i.e., measuring audiometric thresholds in normal hearing participants in a sound-attenuated room, the results provide first evidence that the mobile self-assessment screening audiometer with calibrated headphones is measuring hearing thresholds, which are comparable to those obtained by a standard automated audiometer. Thus, the app could serve as a valid screening audiometer. Future studies could explore the validity of the app for patients with hearing loss or for children.

## Data Availability Statement

The datasets generated for this study are available on request to the corresponding author.

## Ethics Statement

This research was conducted in conformity to the regulations and ethical standards of the Hamburg Medical Association (Ärztekammer Hamburg). The ethical review board of the Hamburg Medical Association waived the requirement for ethical approval for this study after screening the study protocol given that the experimental execution and data handling did not meet the criteria for an obligatory ethical review according to national guidelines and local legislation. Data storage and analysis were fully anonymized. Written informed consent was collected by all participants before participation according to the Declaration of Helsinki.

## Author Contributions

TS and GS conceived and designed the study. AC performed the testing. AC, TS, and GS analyzed the data. JN contributed materials or analysis tools. AC and TS wrote the manuscript. All authors discussed the results, edited, and approved the manuscript.

## Conflict of Interest

GS and JN received reimbursements or salary from Sonormed GmbH (Hamburg, Germany) to participate in the development of the app-based hearing test evaluated in the current study. JN received salary from Mindo Software SLU (Barcelona, Spain). The company was involved in the software development of the app. The remaining authors declare that the research was conducted in the absence of any commercial or financial relationships that could be construed as a potential conflict of interest.
