# Supplementary material for: Evaluation of Accuracy and Reliability of a Mobile Screening Audiometer in Normal Hearing Adults
Source: Front Psychol. 2020 Apr 29;11:744. doi: 10.3389/fpsyg.2020.00744 (PMC7201107; doi:10.3389/fpsyg.2020.00744)
Supplement: Supplementary file 1 [file Presentation_1.pdf]

Calibration Procedure

This document describes the calibration procedure of the iPad-based hearing screening device Audimatch. Audimatch is developed and marketed by Sonormed GmbH.

Location of the calibration

The calibration of the hardware is executed by Sonormed and a professional and certified calibration company.

Headphone Calibration

Purpose

The sound pressure level produced by the hearing screening device's headphones is calibrated with a B&K 2260 Observer in order to assure that the correct sound levels are presented during the hearing test. The calibration is realised in dB HL (hearing level):

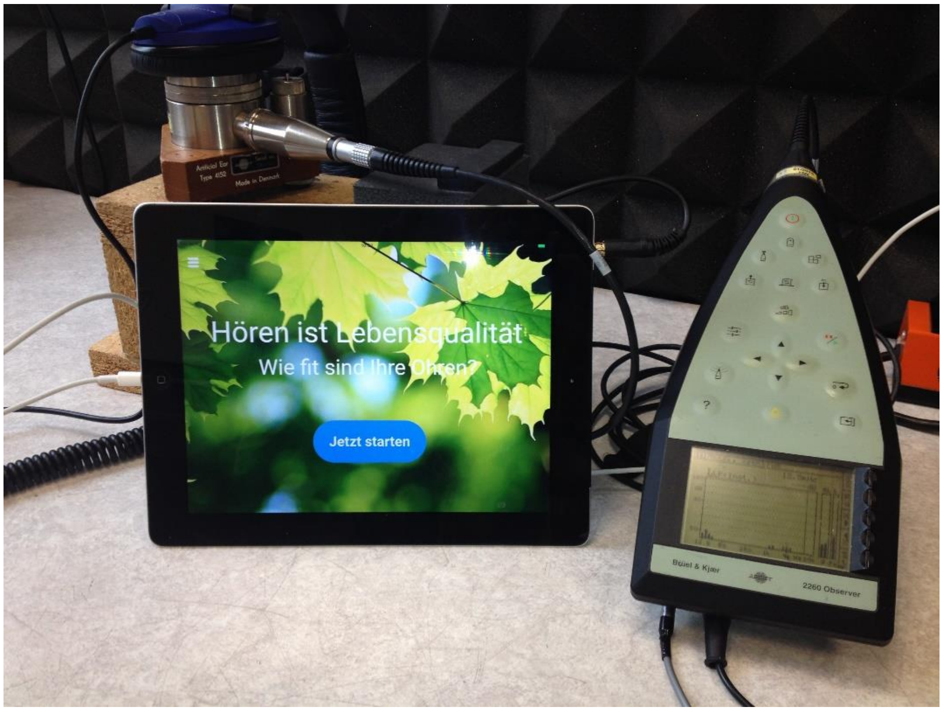

Method

The headphone calibration is executed by a professional and certified calibration company.

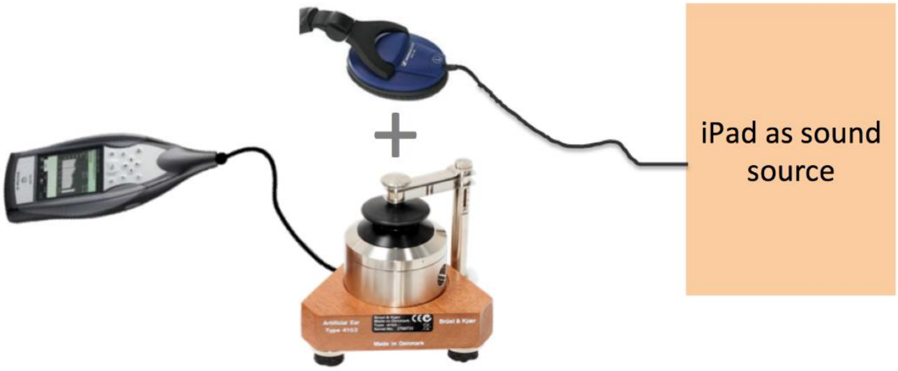

Block diagram of the headphone calibration: the iPad is connected to the headphone and the output level of the headphone is adjusted in the iPad by software to reach 70 dB HL on the artificial mastoid with sound level meter.

The measurement is executed at 125, 250, 500, 750, 1000, 1500, 2000, 3000, 4000, 6000, 8000 and 12000 Hz. At each frequency, the device is adjusted so that the calibrated sound level meter (on the left in the above figure) outputs a target sound hearing level of 70 dB HL.

If the sound output fails to reach 70 dB HL at one or more frequencies, the calibration cannot be executed and Sonormed needs to be contacted.

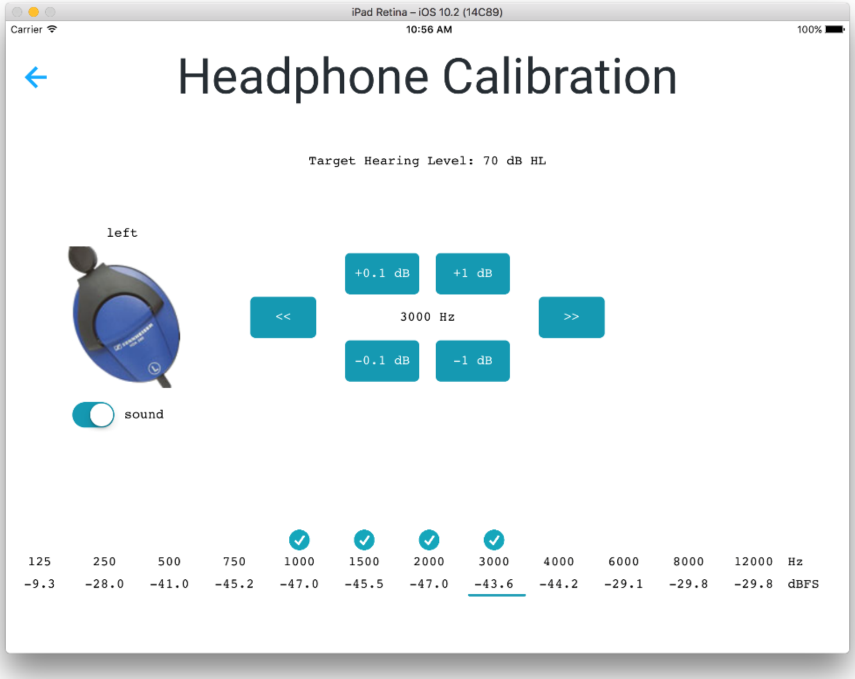

Screen of the device for headphone calibration (left side): the iPad sound level is increased or decreased to reach 70 dB HL in the sound level meter

Total Harmonic Distortion

Purpose

At maximal sound pressure level, the headphone should be able to output pure tones without creating more than 2.5 % of harmonic distortion at the levels defined in IEC 60645-1:2001.

Method

The measurement of the total harmonic distortion is executed by a professional and certified calibration company according to IEC 60645-1:

Table 3 – Maximum permissible acoustic total harmonic distortion, expressed in percentage of sound pressure or vibratory force for supra-aural, circumaural, insert earphones and bone vibrators

	Air conduction			Bone conduction		
Frequency range (Hz)	125 to 250	315 to 400	500 to 5 000	250 to 400	500 to 800	1 000 to 4 000
Hearing level[a] (dB)	75	90	110	20	50	60
Total harmonic distortion (%)	2,5	2,5	2,5	5,5	5,5	5,5

[a] Or maximum output level of the audiometer, whichever is lower. For circumaural and insert earphones the hearing level shall be 10 dB less than the levels specified in the table.

Section 6.1.3 of IEC 60645-1:2001 Electroacoustics — Audiological equipment — Part 1: Pure-tone audiometers

The device offers a screen that outputs the maximal Hearing Level (dB HL) and sound pressure level (dB SPL) as well as A-Weighted Sound Pressure level (dBA)
at 125, 250, 500, 750, 1000, 1500, 2000, 3000, 4000, 6000, and 8000 Hz:

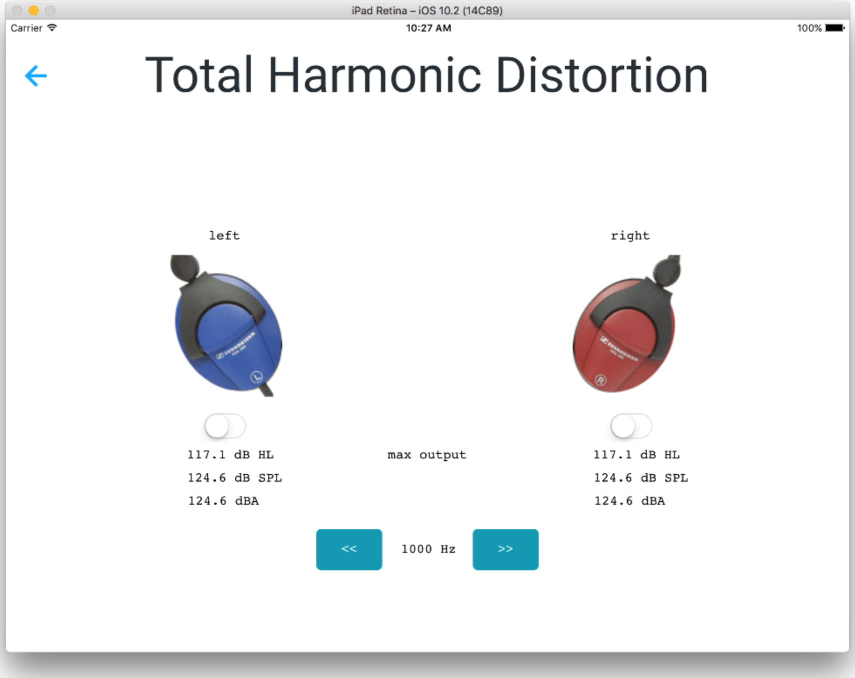

Screen of the device for the measurement of total harmonic distortion

Linearity

Purpose

The reduction or increase of the sound pressure level by changing the amplitude of the digital signal in the device shall correspond to the same reduction or increase of the acoustical sound pressure produced by the headphones.

Method

The measurement of linearity is executed by a professional and certified calibration company. For this measurement the device allows to present tones with 10 dB difference in the sound pressure level:

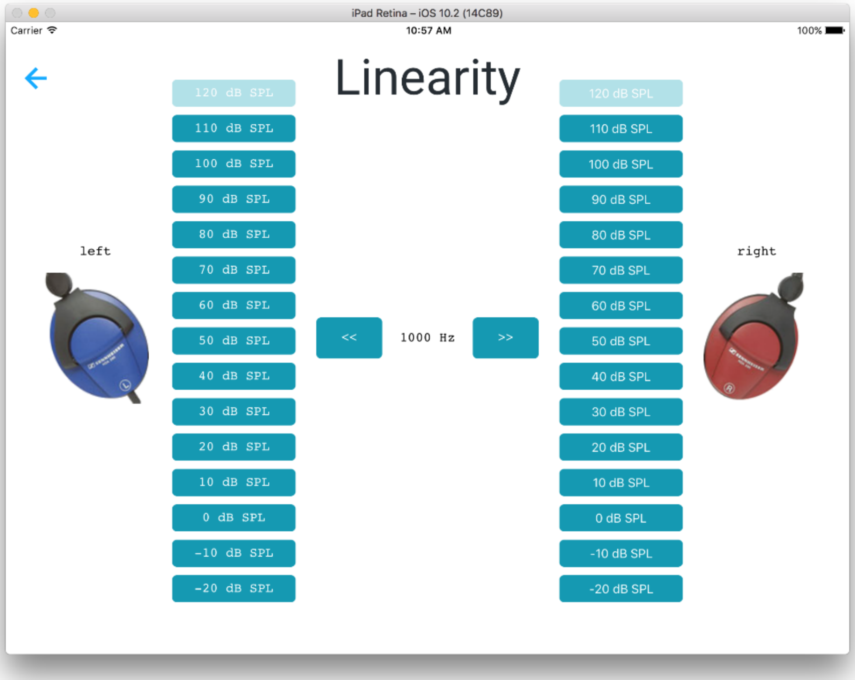

The corresponding screen of the device for measuring its linearity

Microphone Calibration

Purpose

The hearing screening device's microphone must be calibrated because it is used to measure the sound level in the hearing test room to determine if the sound level is low enough to execute a hearing test according to ANSI-ASA standard S3.1-1999 "Maximum Permissible Ambient Noise Levels for Audiometric Test Rooms".

Method

The calibration of the device's microphone is executed at Sonormed by a qualified employee with a technical university degree. In order to lower the effect of standing waves that could influence the sound pressure of pure tones depending on slight differences of the device's physical location, wobble tones are used for the calibration of the device's microphone.

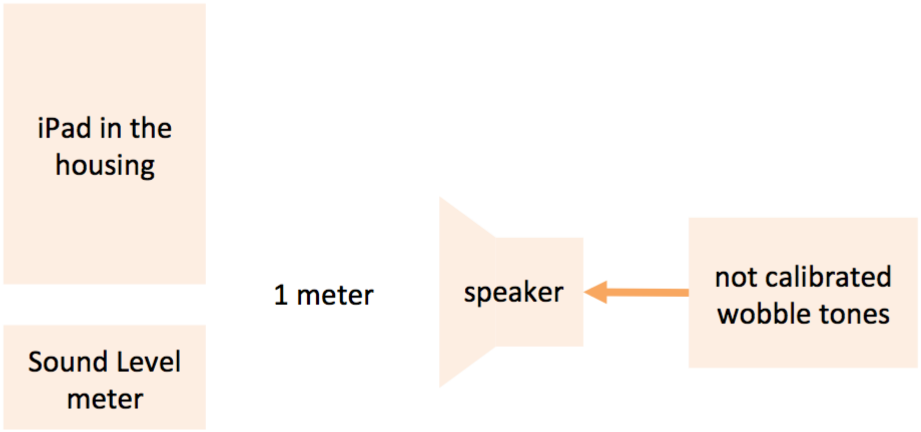

Block diagram of the microphone calibration

The wobble tones were generated with the Audacity software on a Laptop using the following nyquist prompt commands, which result in wobble tones that are centred at the frequencies 125, 250, 500, 750, 1000, 1500, 2000, 3000, 4000, 6000, and 8000 Hz on a logarithmic scale. The lower centre and upper frequencies of the wobble tones are listed as wobblelow and wobbleup below, in addition to the parameters and nyquist commands used in audacity:

lower	wobblelow	center	wobbleup	upper	AudacityCenter	AudacityWidth
89	107	125	151	177	129	22
177	213	250	302	354	258	44
354	427	500	556	612	491	65
612	681	750	808	866	745	63
866	933	1000	1112	1225	1023	90
1225	1362	1500	1616	1732	1489	127
1732	1866	2000	2225	2449	2045	179
2449	2725	3000	3232	3464	2978	254
3464	3732	4000	4449	4899	4091	359
4899	5449	6000	6464	6928	5957	507
6928	7464	8000	8899	9798	8182	717
9798	10899	12000	13655	15310	12277	1378

```
use this to generate the wobble tones in Audacity:
(hzosc (sum       129 (mult       22 (hzosc 5))))
(hzosc (sum       258 (mult       44 (hzosc 5))))
(hzosc (sum       491 (mult       65 (hzosc 5))))
(hzosc (sum       745 (mult       63 (hzosc 5))))
(hzosc (sum      1023 (mult       90 (hzosc 5))))
(hzosc (sum      1489 (mult      127 (hzosc 5))))
(hzosc (sum      2045 (mult      179 (hzosc 5))))
(hzosc (sum      2978 (mult      254 (hzosc 5))))
(hzosc (sum      4091 (mult      359 (hzosc 5))))
(hzosc (sum      5957 (mult      507 (hzosc 5))))
(hzosc (sum      8182 (mult      717 (hzosc 5))))
(hzosc (sum     12277 (mult     1378 (hzosc 5))))
```

The sound level meter is a Voltcraft SL-100, which has been calibrated by a professional and certified calibration company (see below). The speaker used to present the narrow band noise is Creative Soundblaster Roar 2 (S/N: YKMF8190550R00949X). The device is set in the same housing that is used for the hearing test. The microphone of the sound level meter is positioned as close as possible to the microphone of the device and is oriented in the same direction.

During the microphone calibration, a narrow wobble tone is presented with centre frequencies of 125, 250, 500, 750, 1000, 1500, 2000, 3000, 4000, 6000, and 8000 Hz. The wobble tone at 12000 Hz is not used in this calibration procedure.

The calibration of the device’s microphone is challenged by the dynamic nature of the wobble tones and by possible sounds in the environment. The calibration is therefore executed in a very silent room.

During the calibration of the device’s microphone, its sensitivity is adjusted using the device’s "+ sensitive" and "- sensitive" buttons so that the sound level in dB SPL (which is displayed between these buttons) matches the sound level displayed on the sound level meter:

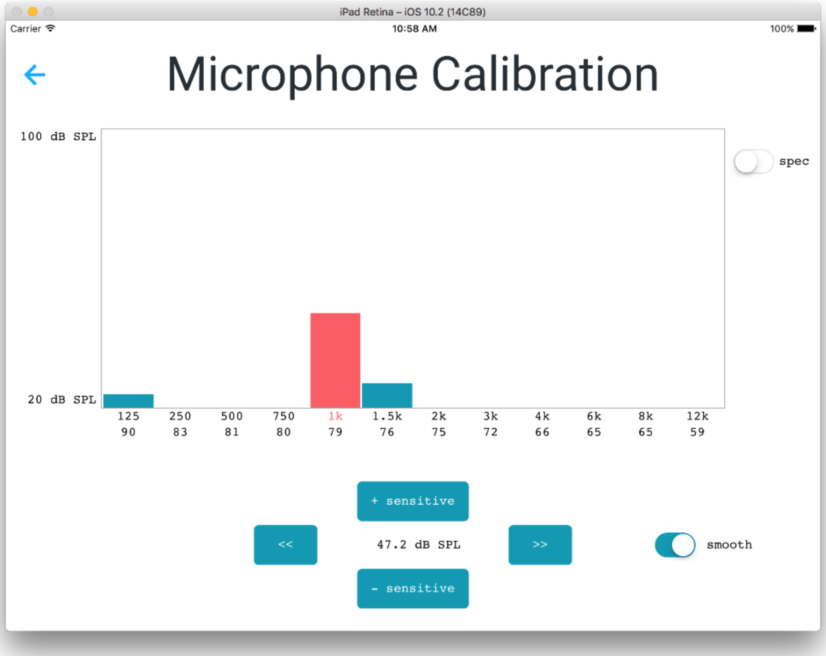

Screen of the device for the microphone calibration

Procedure

1. Prepare a computer with the wobble tone signals and a speaker.
    1. Remove the black foam cover and set the sound level meter up:
       (Turn every setting off and on again to be sure the sound level meter saved the right setting)
    2. C-weighting
    3. Low sensitivity (LO)
    4. Slow (S)

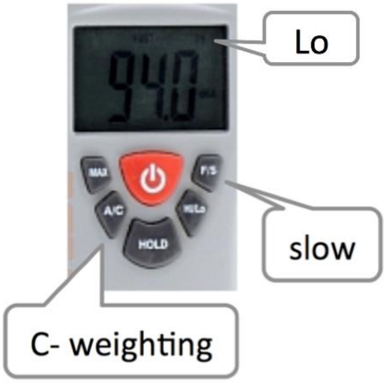

2. Position the sound level meter and the hearing screening device's microphone in its housing close to each other at a distance of about 1 meter from the speakers.
3. Play the wobble tone signals one-by-one and adjust the sound level at the computer and/or speaker so that the measured sound level at the sound level meter is between 60 and 80 dB SPL.
   (In case of a calibrated but not adjusted sound level meter: Don't forget to add calibrated correction values of the SPL meter to the read measurements.)
4. Use the Microphone calibration screen on the device to adjust its microphone sensitivity to read the same sound level as the value given on the sound level meter, within the uncertainty of the fluctuating values. The desired precision is 2 dB. Write down the results.
   (In case of a calibrated but not adjusted sound level meter: Don't forget to add calibrated correction values of the SPL meter to the read measurements.)
5. Store the calibration result.
6. Write the microphone calibration report. It should contain:
    1. The name of the person conducting the calibration
    2. Date and place
    3. Serial number of the Voltcraft SL-100 sound pressure meter
    4. Date and operator's name of last calibration of the sound level meter
    5. Readout of SPL-meter and corresponding readout of sound level and sensitivity level of calibrated and adjusted hearing screening device unit comprising app, iPad and headphones
